# The susceptibility of T5-TG12 of the *CFTR* gene in chronic bronchitis occurrence in a Chinese population in Jiangsu province, China

**DOI:** 10.7555/JBR.26.20120015

**Published:** 2012-04-22

**Authors:** Ping Wang, Satoru Naruse, Hong Yin, Zhongfang Yu, Tianqu Zhuang, Wei Ding, Yanmin Wu, Muxin Wei

**Affiliations:** a Department of Traditional Chinese Medicine, the First Affiliated Hospital, Nanjing Medical University, Nanjing, Jiangsu 210029, China;; b Department of Internal Medicine, the Miyoshi-City Hospital, Nagoya 466-8550, Japan;; c Department of Internal Medicine, the Affiliated Yangzhou Hospital, Traditional Chinese Medicine of the Nanjing University of Chinese Medicine, Yangzhou, Jiangsu 225009, China;; d Department of Paediatrics, Yixing Hospital, Traditional Chinese Medicine, Yixing, Jiangsu 214200, China;; e Department of Andrology, the Affiliated Suzhou Hospital, Traditional Chinese Medicine of the Nanjing University of Chinese Medicine, Suzhou, Jiangsu 215003, China.

**Keywords:** cystic fibrosis transmembrane conductance regulator (CFTR), T5-TG12, chronic bronchitis, Chinese population

## Abstract

Mutations in the cystic fibrosis transmembrane conductance regulator (*CFTR*) gene have been implicated in the onset of cystic fibrosis and other clinical respiratory disorders. In the present study, we investigated the role of *CFTR* variations, poly-T, TG-repeats, and M470V in susceptibility to bronchial asthma and chronic bronchitis in a Chinese population in Jiangsu province, China. A total of 72 bronchial asthma patients, 68 chronic bronchitis patients, and 117 healthy subjects were included in this study. The Tn-TGm haplotype was sequenced and the *CFTR* variant M470V was detected using restriction fragment length polymorphism (RFLP). We found that the frequency of T5-TG12-V470 in chronic bronchitis patients was 0.07%, which was notably higher than that in healthy subjects (0.01%) and bronchial asthma patients (0.04%). Thus, the presence of the T5-TG12 haplotype of the *CFTR* gene is likely to play a role in the development and progression of respiratory conditions, such as chronic bronchitis.

## INTRODUCTION

The cystic fibrosis transmembrane conductance regulator (CFTR) protein, which is 1,480 amino acids long and has a molecular weight of 168,173 Da, is a cAMP-activated chloride (Cl-) channel located on the apical membranes of epithelial cells. In many tissues, such as the airways, sweat glands, pancreas cells, bile ducts and genital ducts, the CFTR protein contributes to the regulation of normal physiological functions[1],[2]. Mutations in the gene encoding CFTR may cause cystic fibrosis (CF), a common autosomal recessive disease affecting 1/2,000-4,000 newborns in the Caucasian populations. Defective chloride ion transport generated by these mutations may lead to dehydrated secretions that contribute to chronic lung infection, gastrointestinal tract alterations, and male infertility[3]. Currently, 1,903 characterized mutations are listed in the database of *CFTR* gene mutations (http://www.genet.sickkids.on.ca/cftr/StatisticsPage.html). Though CF is one of the most severe manifestations of *CFTR* gene mutation, other respiratory conditions may also occur, such as bronchial asthma and chronic bronchitis. In CF, severe obstruction of the columnar epithelial tissues produces rich mucin secretions that result in the absence of functional CFTR proteins in many tissues. This effect leads to pathological phenomena characterized by increased airway resistance, elevated sputum production, excessive cough, and chronic susceptibility to infectious bronchitis[4],[5].

Bronchial asthma is another chronic inflammatory lung disease characterized by variable airflow obstruction and bronchial hyperresponsiveness occurring in paroxysms and often related to specified triggering events. Dysfunction in the *CFTR* gene may lead to this disease, though environmental factors such as cigarette smoking are also often triggers for attacks. Similarly, chronic bronchitis is a syndrome defined by chronic sputum production and airway inflammatory processes that involve the epithelium, subepithelium, bronchial glands, and bronchial smooth muscle[6],[7]. Like bronchial asthma, chronic bronchitis can also be worsened by environmental factors. Cigarette smoking has been implicated in the onset of chronic bronchitis through its role in impairing CFTR protein function, posing significantly elevated risks to the people who are genetically predisposed to chronic bronchitis[8]. The relationship between cigarette smoking and respiratory conditions has been widely explored in scientific literature, yielding a variety of results that indicate both environmental and genetic roles in the onset of bronchial asthma and chronic bronchitis.

Owing to polymorphisms in its genetic component, the prevalence of CF and other respiratory conditions varies significantly among different populations, with CF being quite common in the Caucasian populations but very rare in the Asian populations. The polymorphisms known as poly-T, TG-repeats, and M470V have been well documented for their roles in the development and predisposition to CF and related respiratory diseases. The poly-T tract is located at the junction of intron 8 (IVS-8) and exon 9 and influences transcription processes, thereby reducing the amount of normal CFTR protein. TG-repeat polymorphisms located on the 5′ portion of the poly-T influence the splicing of exon 9. In particular, the TGm locus influences the penetrance of the T5 allele, producing a non-functional CFTR protein and variable CF symptoms[9]. The M470V polymorphism in exon 10 affects intrinsic chloride activity, thereby indirectly affecting the function of the CFTR protein[10]. Previous studies suggested that when the IVS-8-5T allele of the *CFTR* gene was combined with higher numbers of TGm and the V470 variant, a notably higher morbidity rate was observed in patients with a related genetically-linked condition known as congenital bilateral absence of the vas deferens (CBAVD)[11]. Based on these findings, the phenotypic expression of Tn-TGm-M470V in the *CFTR* gene may have similar relevance to respiratory conditions, such as chronic bronchitis and bronchial asthma.

Previous study has yielded critical information related to the polymorphism of poly-T, TG-repeats, and M470V in healthy Chinese populations[12]. In an effort to expand the scope of these observations, the present study was to investigate the specific impact of poly-T, TG-repeats, and M470V CFTR variations on susceptibility of a representative Chinese Han population in Jiangsu Province to bronchial asthma and chronic bronchitis. Furthermore, the present study reaffirmed the relationship between the Tn-TGm-M470V haplotype and environmental factors such as cigarette smoking in bronchial asthma and chronic bronchitis patients.

## SUBJECTS AND METHODS

### Subjects

A total of 140 subjects were treated or observed at one of the three hospitals in Jiangsu Province (the First Affiliated Hospital of Nanjing Medical University, Yangzhou Hospital of Traditional Chinese Medicine and Yixing Hospital of Traditional Chinese Medicine) between September 2007 and December 2010. Informed consent was obtained from each subject. The study protocol was approved by the local institutional review boards at the authors' affiliated institutions. The primary experiments were performed at the First Affiliated Hospital of Nanjing Medical University. The diagnosis of bronchial asthma and chronic bronchitis was performed according to the Guideline of Asthma Treatment and Prevention in China (GATPC, 2003)[13] published by the asthma group of the Chinese Medical Association (CMA) of Respiratory Diseases and Internal Medicine[14]. Patients with additional abnormalities or complicating conditions, such as respiratory tract neoplasm and pulmonary tuberculosis, were excluded from the study. Clinical profiles and backgrounds of all patients were extracted from medical records and direct interviews conducted by medical staff. Poly-T, TG-repeats, and M470V polymorphisms testing was conducted in the samples collected from the 140 study subjects and additionally from 117 healthy volunteers without respiratory symptoms, serving as an experimental control.

### Genotyping

Genomic DNA was extracted from whole blood samples using the QIAamp® DNA blood mini kit (Qiagen, Hilden, Germany). Polymerase chain reaction (PCR) was carried out by the Ex *Taq* polymerase (TaKaRa, Japan) with a GeneAmp PCR system (Model PTC-200, Bio-Red, Foster City, CA, USA). The following PCR protocol was used: 94°C for 5 min, 94°C for 30 s×35 cycles, 60°C for 60 s, 72°C for 30 s, and 72°C for 10 min. The following primers were used: intron 8 and exon 9 junction with 5′-CCATGTGCTTTTCAAACTAATTGT-3′ and 5′-TAAAGTTATTGAATGCTC GCCATG-3′, and exon 10 with 5′-TTGTGCATAGCAGAGTACCTGAAA-3′ and 5′-GCT TCTTAAAGCATAGGTCATGTG-3′. The 506-bp PCR product was purified using the High Pure PCR Product Purification Kit (Roche Diagnostics, Mannheim, Germany) and subjected to sequencing by an automated sequencer ABI737 from in Invitrogen (Shanghai, China). Each sample was tested twice. The haplotypes of poly-T TG repeats were confirmed by the DNA sequencing results. Restriction fragment length polymorphism (RFLP) with *Hph*I was used to detect M470V polymorphism by garose gel electrophoresis (AGE) images of the pure PCR products. The frequency of each haplotype of TG-repeats and M470V was analyzed by the equation derived from the Hardy-Weinberg Law.

### Statistical analysis

Descriptive statistics for all variables were examined for all the three groups and among smokers and nonsmokers. Qualitative data in multiple groups were analyzed by one-way analysis of variance (One-way ANOVA). Differences between two groups were examined using Student-Newman-Keuls (SNK) test. The frequency of each haplotype of TG-repeats and M470V was analyzed by the following equation derived from the Hardy-Weinberg Law: (P_1_ + P_2_ + P_3_ +.+ P_m_)^2^ = 1, where P_1_ + P_2_ + P_3_ + ... + P_m_ = 1, and P_1_^2^, P_2_^2^, P_3_^2^, P_m_^2^ are the frequencies of homozygosity for either locus or both loci. Haplotypes consisting of the three loci poly-T, TG-repeats, and M470V polymorphisms were analyzed by Chi-square test. When an expected cell value was less than 5, Fisher's exact test was applied. All *P*-values were based on two-sided comparisons, and a *P*-value of less than 0.05 was considered statistically significant.

## RESULTS

### Demographic characteristics of study subjects

The characteristics of age, gender, and cigarette smoking habits of the 140 fully affected subjects and 117 healthy control subjects are shown in ***Table 1***. These subjects were divided into three groups: the bronchial asthma group (*n* = 72, range, 19-72 years), chronic bronchitis group (*n* = 68, range, 35-75 years), and control group (*n* = 117, range 18-68 years). The average age of males and females had no statistical significance within each group. Smoking history was additionally collected from all subjects.

### Poly-T, TG-repeats, and M470V

The occurrence of the T7, T5, T9 and T6 alleles, in the order of occurrence frequency, was recorded for each group, as shown in ***Table 2***. The T7 allele frequency in the chronic bronchitis group was lower (88.2%) than that observed in the control group (93.6%) or the bronchial asthma group (89.6%). The T5 allele was the second most common haplotype observed in patients of the present study, with a frequency of 4.3% in the control group, 7.6% in the asthma group and 8.8% in the chronic bronchitis group. The T9 allele was very rare in the control group (0.9%) and slightly more common in the chronic bronchitis group (1.5%). Notably, the T9 allele was completely absent in the bronchial asthma group. The T6 alleles were found in two control subjects and three affected subjects (2 bronchial asthma patients and 1 chronic bronchitis patient).

**Table 1:** Characteristics of 140 patients with bronchial asthma or chronic bronchitis and 117 healthy control subjects

Group	*n* (male/female)	Age		Smokers		Nonsmokers	
		Male	Female	*n* (male/female)	Age	*n* (male/female)	Age
Control	117 (53/64)	31.8±15.9	29.1±11.9	11 (11/0)	51.7±9.0	106 (42/64)	28.1±12.5
Bronchial asthma	072 (39/33)	59.5±10.9	45.5±16.2	3 (3/0)	56.7±7.8	069 (36/33)	52.9±15.5
Chronic bronchitis	068 (40/28)	59.3±8.5	61.8±7.3	25 (20/5)	62.1±8.3	043 (20/23)	59.3±7.9

Age is expressed as mean±SD for all subjects. The average age of males and females has no statistical significance within each group. Smoker: an adult (≥18y) who smokes at least 10 cigarettes per day in the past 2 years.

**Table 2:** Allele frequencies of poly-T, TG-repeats and M470V in 140 patients with bronchial asthma or chronic bronchitis and 117 healthy control subjects

Group	Alleles of poly-T				Alleles of TG-repeats				Alleles of M470V	
	T5	T6	T7	T9	TG10	TG11	TG12	TG13	M	V
Control (2*n* = 234)	10(4.3)	3(1.3)	219(93.6)	2(0.9)	1(0.43)	139(59.4)	94(40.2)	0(0.0)	103(44.0)	131(56.0)
Bronchial asthma (2*n* = 144)	11(7.6)	4(2.8)	129(89.6)	0(0.0)	0(0.0)0	080(55.6)	62(43.1)	2(1.4)	064(44.4)	080(55.6)
Chronic bronchitis (2*n* = 136)	12(8.8)	2(1.5)	120(88.2)	2(1.5)	0(0.0)0	071(52.2)	63(46.3)	2(1.5)	053(39.0)	083(61.0)

There are four alleles (T5, T6, T7 and T9) depending on the number of thymidines in the poly-T tract, and there are four alleles (TG10, TG11, TG12 and TG13) depending on the number of tandem TG repeats adjacent to the poly-T tract in intron 8 of the *CFTR* gene. M470V is coded by a non-synonymous single nucleotide polymorphism in exon 10 of the *CFTR* gene. 2n is the total number of alleles (n is the total number of subjects, each individual has two alleles).

[*n*(%)]

The TG11 and TG12 alleles accounted for more than 95% of total mutations in both affected and control subjects, also shown in ***Table 2***. The frequency distribution of haplotype TG11 was 59.4%, 55.6% and 52.2% in the control group, bronchial asthma group, and chronic bronchitis group, respectively. Two alleles of TG13 were found in the affected groups. The V allele (57.2%) of the M470V gene was slightly more frequent than the M allele (42.8%) in all subjects, as shown in ***Table 2***. The frequency of the V allele in the chronic bronchitis group (61.0%) was not significantly different from that observed in the control group (56.0%, *P* = 0.38) and the bronchial asthma group (55.6%, *P* = 0.39).

### Tn-TGm

The T7-TG11 allele was the major haplotype in all controls (57.7%) and affected subjects (55.6% in bronchial asthma and 50.4% in chronic bronchitis), as shown in ***Table 3***. The frequency of the T5-TG12 allele in the bronchial asthma (7.6%, *P* < 0.05) was significantly higher than that of the control group (3.0%).

### Tn-TGm-M470V

T7-TG11-V470 was the primary haplotype observed ubiquitously throughout the study, with a frequency of 36.8% in the control group, 35.4% in the bronchial asthma group, and 34.6% in the chronic bronchitis group. Frequencies of T7-TG11-M470, T7-TG12-M470, and T7-TG12-V470 were similar in the three groups. The frequency of T5-TG12-V470 in chronic bronchitis (6.6%) was significantly higher than that observed in the control group (1.3%, *P* < 0.05). Furthermore, the T5-TG12-M470 haplotype in the asthma group (3.5%) was more common than that observed in both the chronic bronchitis group (0.7%) and the control group (1.7%).

Cigarette smoking had a significant impact on the diversity of each group. Among the 106 non-smokers in the control group, the haplotype T5-TG12-V470 was observed in only 1 individual (0.9%). Of the 25 patients with confirmed cigarette smoking and chronic bronchitis, the ratio of this haplotype was 18% (9 patients). The haplotype T7-TG11-M/V470 in the 106 non-smoking subjects was more common than that in smoking subjects with chronic bronchitis (***Table 4***), suggesting that smoking does, in fact, play a role in the onset of respiratory conditions in those genetically predisposed to such conditions.

### The gengtype of T5/T5, T5/T6 and T5/T7

Based on the observation that the occurrence of the T5-TG12 haplotype varies significantly between the control, bronchial asthma, and chronic bronchitis groups, the poly-T homozygous frequency was analyzed (***Table 5***). The frequency of the T5/T7 heterozygote was virtually identical for all groups, and the T7/T7 homozygous frequency was observed to be the primary genotype. The proportion of the T5/T5 homozygotes reached 7.4% in the chronic bronchitis group and 6.9% in the asthma group, while the proportion in the control group remained notably low (4.3%). Furthermore, T5/T5 was observed in 4 chronic bronchitis patients with confirmed cigarette smoking (16%) and only 1 non-smoking patient.

**Table 3:** Haplotype and frequencies of IVS-8 Tn-TGm in 140 patients with bronchial asthma or chronic bronchitis and 117 healthy control subjects

Group	T5-TGm			T6-TGm	T7-TGm		T8-TGm
	T5-TG11	T5-TG12	T5-TG13	T6-TG12	T7-TG11	T7-TG12	T9-TG11
Control (2*n* = 234)	3 (1.3)	07 (3.0)^a^0	0 (0.0)	3 (1.3)	135 (57.7)	84 (35.9)	1 (0.4)
Bronchial asthma (2*n* = 144)	0 (0.0)	11 (7.6)^a^0	0 (0.0)	4 (2.8)	080 (55.6)	47 (32.6)	0 (0.0)
Chronic bronchitis (2*n* = 136)	0 (0.0)	10 (7.35)^a^	2 (1.5)	2 (1.5)	069 (50.7)	51 (37.5)	2 (1.5)

There are seven haplotypes of IVS-8 Tn-TGm of the *CFTR* gene which were observed in the present study. 2*n* is the total number of alleles (*n* is the total number of subjects; each individual has two alleles). ^a^*P* < 0.05 *vs* the control group.

[*n*(%)]

**Table 4:** Frequencies of T5/T6-TGm-M470V in smokers and non-smokers

Group	T5-TG11-M	T5-TG12-M	T5-TG12-V	T6-TG12-M	T6-TG12-V	T7-TG11-M	T7-TG11-V	T7-TG12-M	T7-TG12-V
Control									
Smokers (*n* = 11)	0 (0.0)	0 (0.0)0	1 (4.5)0	0 (0.0)	0 (0.0)0	04 (18.2)	05 (22.7)	06 (27.3)	06 (27.3)
Non-smokers (*n* = 106)	3 (1.4)	4 (1.9)0	2 (0.9)0	1 (0.5)	2 (0.9)0	45 (21.2)	81 (38.2)	39 (18.4)	33 (15.6)
Bronchial asthma									
Smokers (*n* = 3)	0 (0.0)	2 (33.3)	0 (0.0)0	0 (0.0)	2 (33.3)	0 (0.0)	0 (0.0)	02 (33.3)	0 (0.0)
Non-smokers (*n* = 69)	0 (0.0)	3 (2.2)0	6 (4.3)0	2 (1.4)	0 (0.0)0	29 (21.0)	51 (37.0)	24 (17.4)	21 (15.2)
Chronic bronchitis									
Smokers (*n* = 25)	0 (0.0)	1 (2.0)0	9 (18.0)	2 (4.0)	0 (0.0)0	06 (12.0)	16 (32.0)	08 (16.0)	06 (12.0)
Non-smokers (*n* = 43)	0 (0.0)	0 (0.0)0	0 (0.0)0	0 (0.0)	0 (0.0)0	16 (18.6)	31 (36.0)	20 (23.3)	17 (19.8)

Smoker: an adult (≥18y) who smokes at least 10 cigarettes per day in the past 2 years. The frequencies of T5/T6-TGm-M470V were observed among the control group, the bronchial asthma group, and the chronic bronchitis group. Frequencies of other haplotypes in smokers and non-smokers: T9-TG10-V in 1 (0.5%) and T9-TG11-M in 1 (0.5%) in the control group non-smokers; T7-TG13-M in 2 (1.4%) in bronchial asthma non-smokers; T9-TG11-V in 2 (4.0%) in chronic bronchitis smokers; T5-TG13-V in 2 (2.3%) in chronic bronchitis non-smokers.

## DISCUSSION

The functional polymorphisms of the *CFTR* gene and resultant CFTR protein previously demonstrated in healthy Chinese populations was further expanded by observation of bronchial asthma and chronic bronchitis in a specific Chinese population in Jiangsu province. The results of the analysis of three previously determined polymorphic loci by frequency of alleles was consistent with previous reports of the frequencies of poly-T, TG-repeats, and M470V CFTR mutations in healthy Chinese populations[12]. These findings are also supported by a multitude of previous studies that suggest that while the frequency of these polymorphisms was quite different in the Caucasian populations, little variation was found in the East Asian populations[15]–[17].

The majority of Tn-TGm haplotypes observed in the present study were T7-TG11 and T7-TG12. The frequency of T7-TG11 in the control group was slightly higher than that in the affected groups, while the frequency of T7-TG12 was notably higher in the chronic bronchitis group. These results are consistent with the findings reporting the T7-TG11 haplotype in normal lung function in a Norwegian asthma population, revealing that the T7-TG11 haplotype may play a similar function in other populations[18]. Furthermore, a linkage study conducted in Iceland confirmed that no interaction exists between CFTR and asthma occurrence[19]. Conversely, the studies in Greece, Denmark, and Spain have reported a positive association between asthma and CF that may represent a genetic linkage between the two conditions[20]–[22]. Based on these findings, there is likely to be no significant expression of T7-TG11 in Chinese bronchial asthma patients; however, additional larger scale studies should be performed to confirm these findings in the East Asian populations.

**Table 5:** The genotype of T5/T5-TGm-M470V, T5/T6-TGm-M470V and T5/T7-TGm-M470V

Genotype	Control		Bronchial asthma		Chronic bronchitis	
	Smokers (*n* = 11) (%)	Non-smokers (*n* = 106) (%)	Smokers (*n* = 3) (%)	Non-smokers (*n* = 69) (%)	Smokers (*n* = 25) (%)	Non-smokers (*n* = 43) (%)
T5-TG11-M/T5-TG11-M		1(0.9)				
T5-TG12-M/T5-TG12-M		2(1.9)	1(33.3)	1(1.4)		
T5-TG12-V/T5-TG12-V		1(0.9)		3(4.3)	4(16.0)	
T5-TG13-V/T5-TG13-V						1(2.3)
T5-TG11-M/T5-TG12-M	1(9.1)					
T5-TG12-V/T7-TG11-V	1(9.1)				1(4.0)	
T5-TG11-M/T6-TG12-M		1(0.9)				
T5-TG12-M/T7-TG11-M				1(1.4)		1(2.3)

Smoker: an adult (≥18 year) who smokes at least 10 cigarettes per day in the past 2 years. There are 8 genotypes of T5-TGm-M470V/Tn-TGm-M470V (*n* = 5, 6 and 7) in this study.

The adverse affects of cigarette smoking on gene expression and tissue function have been well documented and were confirmed by the linkage of smoking to chronic bronchitis in the present study. The T5/T5 genotype was observed in 16% of smoking patients with chronic bronchitis, but was observed in only 2.3% of non-smoking patients with chronic bronchitis. The current study suggested that the T5/T5 genotype is associated with the pathology of chronic bronchitis, though further biochemical studies would be required to elucidate the mechanism underlying the association. Researchers have previously proposed that smoking rapidly impairs the respiratory tissue function by inducing CFTR internalization, leading to dehydration of the airway surface liquid (ASL). The ASL may quickly promote mucus stasis and failure of mucus clearance, resulting in an increased risk of developing chronic bronchitis in affected smokers[8]. Cigarette smoking is likely to further exacerbate the declining levels of CFTR function, resulting in elevated occurrence of respiratory tract diseases such as chronic bronchitis.

Additional studies have directly linked cigarette smoking with the pathogenesis of asthma and chronic bronchitis[23]. Though the mechanism required further exploration, cigarette smoking has been shown to decrease CFTR expression and function *in vitro*, resulting in acquired CFTR deficiency in the nasal respiratory epithelium as well as other tissues of cigarette smokers. Acquired CFTR deficiency, as asserted by many research groups, may contribute to the physiopathology of cigarette-induced diseases such as chronic bronchitis[24]. In addition, second hand smoke has been shown to adversely affect lung function of individuals with CF. Variations in the gene that causes CF (such as *CFTR*) and a CF-modifier gene (such as *TGF*-*β1*) amplified the negative effects of second hand smoke exposure and may contribute to the onset of chronic conditions based on exposure levels and duration[25]. Both direct and second hand cigarette smoking have been clinically implicated in the function of CFTR in the nasal and respiratory tracts tissues.

The M470V polymorphism is a type of nonsynonymous missense mutation caused by a particular amino acid alteration in exon 10 of the M470V locus. The M470 allele has been implicated in delayed CFTR protein maturation, giving rise to an increased probability of opening a chloride channel, unlike the V470 CFTR protein[26]. The *CFTR* gene has been previously reported for its involvement in asthma and bronchiectasis, in addition to its possible involvement in severe conditions, such as chronic obstructive pulmonary diseases. In the present study, the hyperactive M470 allele was found to be more frequent in the affected groups than that in the control group, indicating that such an association is likely to exist in Chinese populations. Conversely, an European study suggested that variation in the *CFTR* gene was primarily associated with the M470 allele, while the V470 allele showed extended haplotype homozygosity[27]. These findings further highlighted the variation between Caucasian and Eastern Asian populations, as the current study found that the M470 allele was less common than the V470 allele in subjects from Jiangsu Province of China, with the frequency observed in the chronic bronchitis group being only 39.0%. These findings suggested that the frequency of the V470 allele increased if the subjects suffer from chronic bronchitis, though the V allele is virtually always associated with the disease in Chinese Jiangsu population.

The T7-TG11-V470 haplotype was shown to be the dominant haplotype in the subjects of Chinese Jiangsu population in the present study, which are similar to the results obtained in Japanese and other East Asian populations[15]. Accumulating data indicated that the T5-TG12-V470 haplotype had a higher frequency of disease than other genotypes in CBAVD patients. Numerous contemporary studies performed on CBAVD patients have reported that a lower number of Tn could alter the binding of TDP43/TARDBP (tar DNA binding protein) to its specific target in a tissue-specific manner[28]–[30]. Additionally, the haplotype T5 is closely related with male infertility, especially in concurrence with long TGm. Elia has proposed that semen hyperviscosity may be considered a “minimal clinical expression” of CF, with CFTR gene sequence variations constituting the genetic basis of the condition[31]. Haplotype T5 has been demonstrated to impact on CFTR function in tissues, and further reduce CFTR function when combined with TG12. In the cases where the haplotype T5/T5 homozygosity is observed, CFTR function is further affected, reducing the overall functional CFTR available in respiratory tissues. The CFTR protein is suspected to play a role in both spermatogenesis and tubal functionality in males, likely through similar mechanisms as those affecting respiratory tract conditions[32],[33]. The expression of haplotype T5-TG12-V470 was elevated in chronic bronchitis, suggesting that the abnormal function of the respiratory tract is also closely related to this phenotype.

The current study provided a specific focus on the investigation of subjects in Jiangsu province, China. While targeted information on certain populations is necessary, the results of this study may be limited in their applicability to other Chinese and East Asian populations, which may diverge from these trends. However, all participants are of Han Nationality, making it likely that the trend observed in this population will be widely applicable to other Chinese populations based on previous studies of population genetics. In order to confirm the results of the present study, a greater number of subjects taken from a wider geographic area are necessary. Additionally, the entire *CFTR* gene was not screened to fully exclude association with other conditions or genetic linkages. The possible significance of Tn-TGm-M470V haplotypes in respiratory system diseases remains to be ascertained, though the trend of its occurrence is clearly evidenced. Though the elevated frequency of T5-TG12-V470 in chronic bronchitis patients has yet to be fully explained, the relationship between smoking and a higher expression of the genotype is clear. Further research focusing on analysis of pulmonary function, sputum properties, and inflammatory markers in the sputum of chronic bronchitis subjects is the next step in elucidating the impact of this genotype on the onset of respiratory diseases.
